# Syntactic flexibility and planning scope: the effect of verb bias on advance planning during sentence recall

**DOI:** 10.3389/fpsyg.2014.01174

**Published:** 2014-10-20

**Authors:** Maartje van de Velde, Antje S. Meyer

**Affiliations:** ^1^Psychology of Language, Max Planck Institute for PsycholinguisticsNijmegen, Netherlands; ^2^Max Planck International Research School for Language SciencesNijmegen, Netherlands; ^3^Donders Institute for Brain, Cognition and Behavior, Radboud UniversityNijmegen, Netherlands

**Keywords:** syntactic flexibility, language production, advance planning, frequency effects, RSVP paradigm, sentence recall

## Abstract

In sentence production, grammatical advance planning scope depends on contextual factors (e.g., time pressure), linguistic factors (e.g., ease of structural processing), and cognitive factors (e.g., production speed). The present study tests the influence of the availability of multiple syntactic alternatives (i.e., syntactic flexibility) on the scope of advance planning during the recall of Dutch dative phrases. We manipulated syntactic flexibility by using verbs with a strong bias or a weak bias toward one structural alternative in sentence frames accepting both verbs (e.g., strong/weak bias: *De ober schotelt/serveert de klant de maaltijd [voor]* “The waiter dishes out/serves the customer the meal”). To assess lexical planning scope, we varied the frequency of the first post-verbal noun (N1, Experiment 1) or the second post-verbal noun (N2, Experiment 2). In each experiment, 36 speakers produced the verb phrases in a rapid serial visual presentation (RSVP) paradigm. On each trial, they read a sentence presented one word at a time, performed a short distractor task, and then saw a sentence preamble (e.g., *De ober…*) which they had to complete to form the presented sentence. Onset latencies were compared using linear mixed effects models. N1 frequency did not produce any effects. N2 frequency only affected sentence onsets in the weak verb bias condition and especially in slow speakers. These findings highlight the dependency of planning scope during sentence recall on the grammatical properties of the verb and the frequency of post-verbal nouns. Implications for utterance planning in everyday speech are discussed.

## INTRODUCTION

In sentence production, words are retrieved from the mental lexicon and combined into grammatical sequences. This is done at an impressive rate. Dutch speakers have an average conversational speaking rate of 4.23 syllables per second (Verhoeven et al., 2004). According to Indefrey and Levelt (2004) encoding of a single word and preparing the articulation of its first syllable takes 600 ms on average. For sentences to be produced fast and fluently, it is therefore inevitable that part of the speech plan is prepared before speech onset.

Bock and Levelt (1994) proposed that speech planning occurs in three stages. First a message is constructed that specifies the intended meaning of the utterance. In a second stage, the message is grammaticalized via functional and positional encoding processes. Functional encoding comprises the retrieval of lexical concepts and lemmas (i.e., grammatical representations of words) and the assignment of these lemmas to grammatical roles (e.g., the subject role). During positional encoding, lexical items are given a serial order and syntactic structures are built. The third stage is the construction of the sound form of the utterance during phonological encoding. Thus, in this model the constituent structure of sentences is generated in two stages. Other authors have argued for direct, single-stage mapping between the message and the constituent structure (Pickering et al., 2002; Cai et al., 2012). The models differ, among other things, in their predictions about lexical influences on grammatical encoding processes. According to single-stage models, lexical accessibility can directly influence word order–and thus syntactic choices–such that highly accessible (e.g., high frequency) units are prioritized. In contrast, multiple-stage models postulate that the thematic structure of the message is first mapped to a functional-grammatical structure, thereby driving syntactic choice independently of lexical influences (Chang, 2002; Chang et al., 2006).

Earlier research on the advance planning of sentences with varying phrase structures has found evidence for the phrase as the default planning unit, for instance by showing that speakers take longer to initiate sentences starting with complex (e.g., *the dog and the hat move*) than simple noun phrases (e.g., *the dog* *moves*; Smith and Wheeldon, 1999; Martin et al., 2010; Wheeldon et al., 2013). However, in other studies using the same sentence types, lexical planning scopes ranging from single lexical items to entire clauses have been found (Meyer, 1996; Griffin, 2001). The production of more complex sentences (such as descriptions of transitive events) has also proven to be flexible, with speakers sometimes prioritizing the encoding of single linguistic elements and sometimes encoding an abstract plan of the utterance before speaking (Gleitman et al., 2007; Kuchinsky and Bock, 2010; Van de Velde et al., 2014). Together, these findings suggest that planning scope is not fixed but variable.

Several studies have attempted to identify the conditions under which speakers decrease or extend their scope of planning. Ferreira and Swets (2002) examined how time pressure affects grammatical planning scope in two experiments using two-digit sums (e.g., 9 + 7 = ?). Speakers were instructed to formulate their answers to these sums as follows: (a) “sixteen,” (b) “sixteen... is the answer,” or (c) “The answer is... sixteen.” Besides utterance type, the difficulty of the arithmetic problem was manipulated. In a second experiment, participants were prompted to start speaking as quickly as possible through the use of a deadline procedure. Speech onsets and utterance durations were measured. In both experiments, speech onset latencies were similar for all three utterance types but speakers initiated their utterances later when problem difficulty increased. Only in Experiment 2 did utterance *duration* also depend on problem difficulty such that answers to difficult arithmetic sums took longer to formulate. This suggests that speakers planned and spoke simultaneously when they were prompted to start their utterance immediately. In contrast, when there was no pressure to start speaking quickly, speakers made use of more extensive advance planning when generating complex constructions and hence showed longer onset latencies when problem difficulty increased.

In addition to contextual factors, speaker characteristics may also have an influence on the amount of advance planning. Wagner et al. (2010) found large differences in planning scope related to speaking rate. They used a picture-word interference paradigm to measure grammatical planning scope: speakers had to produce simple sentences describing objects on a screen (e.g., *the frog is next to the mug*). Unrelated or semantically related auditory distractors were presented at picture onset. Onset latencies were measured to examine semantic interference effects on the first and second object, which indexed the scope of grammatical encoding. Dividing their sample into a group of slow speakers and a group of fast speakers (based on their average naming latencies in an unrelated distractor condition), they found that slow speakers showed larger interference effects on the second noun, suggesting that they engaged in more advance planning than fast speakers. A similar difference was found by Wheeldon et al. (2013), who used the same type of sentences, but made use of a picture preview to increase the accessibility of the second noun. When it was known which sentence position the previewed object would occupy, slow speakers showed a larger preview benefit than fast speakers. The authors concluded that slow speakers have a larger lexical processing scope than fast speakers.

Planning scope is also sensitive to linguistic factors such as ease of structural assembly. In the study by Wagner et al. (2010) described above, speakers had to produce simple sentences (e.g., *the frog is next to the mug*) and more complex structures (e.g., *the red frog is next to the red mug*). In two of their experiments, speakers only used one sentence type (simple or with adjectives) while in another experiment they had to switch between sentence types. Wagner et al. (2010) found that speakers made use of more parallel planning, showing interference from the semantically related auditory distractors on both the first and the second noun, when they only had to produce one sentence type. Oppermann et al. (2010) showed the same pattern for phonological advance planning. These results suggest that when structures are easy to produce (because they are repeated), lexical planning scope may be expanded. Konopka (2012) tested this hypothesis by comparing the production of sentences beginning with conjoined noun phrases (e.g., *the saw and the ax are above the cup*) after manipulating structural and lexical processing ease using structural priming and a lexical frequency manipulation. Conjoined noun phrases either contained semantically related or unrelated objects. Lexical planning scope was measured as the degree of semantic interference from the first onto the second object. Semantic interference was only found for structurally primed sentences beginning with easily accessible words. This result indicates that speakers engage more in advance planning when sentence structures are easy to assemble than when structures are more difficult to construct. Relatedly, Konopka and Meyer (2014) found that if relational encoding in picture naming (i.e., the encoding of causal relations instead of individual characters) is facilitated through the use of easily apprehensible events and/or priming the to-be-used structure, speakers shift from a piecemeal toward a more parallel planning strategy.

Another linguistic factor that may influence planning scope is syntactic flexibility (Myachykov et al., 2013; see also Ferreira, 1996). Syntactic flexibility refers to the availability of syntactic choices during grammatical encoding. According to some models, syntactic flexibility leads to competition between syntactic alternatives (Ferreira, 1996; Dell et al., 1999; Myachykov et al., 2013; Kempen, 2014). For example, when producing a dative sentence a speaker can choose between a prepositional object (PO, e.g., *Peter gives the apple to Mary*) and a double object (DO, e.g., *Peter gives Mary the apple*) frame. Under some circumstances, structural alternatives may be equipotent, meaning that they are activated to the same degree. At the choice point of the dative sentence, i.e., after the verb (*give*), when a structural alternative is uniquely determined, the grammatical encoding system can either activate the indirect object or the direct object slot. The degree to which the indirect object and direct object are prepared while the system decides which structure to produce is reflected in the planning scope. If both objects are lexically encoded, the lexical advance planning scope is wider than when only one option is prepared.

Myachykov et al. (2013) examined whether the number of available syntactic options used in a picture naming task in one experiment could predict sentence-initial processing load in a second picture naming experiment. In Experiment 1, speakers of English and Russian were encouraged to produce as many structural alternatives for a set of depicted events as possible within 15 s per picture. Since Russian grammar offers more options for building transitive constructions than English grammar, Russian participants were expected to use a wider range of syntactic options than English participants. In Experiment 2, a new group of English and Russian participants described the same set of pictures while their eye-gaze was measured. The goal of the study was to link syntactic flexibility in the first experiment to sentence initial processing load in the second experiment, indexed by sentence onset latency and eye-voice span (i.e., the temporal lag between the offset of the last fixation to a referent and producing that referent’s name). As expected, Russian speakers used a wider range of syntactic alternatives than English speakers. In Experiment 2 they were also slower to initiate their sentences and showed longer eye-voice spans for sentence-initial subject nouns than for object nouns. This eye-voice span pattern suggests a planning strategy involving the (partial) preparation of non-initial increments or an abstract framework before sentence onset (Bock et al., 2003). In contrast, English speakers showed shorter onset latencies and longer eye-voice spans for object nouns than subject nouns, indicative of more incremental production. More importantly, in both groups syntactic flexibility in Experiment 1 predicted sentence-initial latency effects in Experiment 2, indicating that more syntactic alternatives led to longer onset latencies after accounting for the effect of language (i.e., English vs. Russian).

The above findings indicate that syntactic flexibility may lead to a higher sentence-initial processing load and longer eye-voice spans for subject than object nouns. The authors interpret these results as demonstrating competition between syntactic frames. They propose that competition between frames leads to an expansion of planning scope, as a larger part of the syntactic plan needs to be prepared prior to speech onset because of the necessity of making a syntactic choice. However, Myachykov’s study does not provide a direct link between syntactic flexibility and grammatical planning scope; instead the study only shows that syntactic flexibility and sentence initial processing load are linked.

The current study aimed to connect syntactic flexibility and planning scope directly by adding a manipulation of lexical accessibility. The question was whether increased syntactic flexibility lead to increasing planning scope. In an experiment using rapid serial visual presentation (RSVP; Potter and Lombardi, 1990, 1998; Lombardi and Potter, 1992; see below for details) participants produced dative sentences featuring verbs with a bias toward the PO or DO dative. Syntactic flexibility was manipulated by using verbs varying in the strength of bias for one of the structural alternatives (see 1a and 1b).

(1a) *De ober serveert de koning het feestmaal* (weak DO bias).
       “The waiter serves the king the banquet.”
(1b) *De ober schotelt de koning het feestmaal voor* (strong DO bias).
       “The waiter dishes the king the banquet out”
       [“The waiter dishes out the banquet to the king”]

Verb bias is the preference of a verb for a syntactic structure, based on its frequency of co-occurrence with the verb (Stallings et al., 1998; Colleman, 2009). The strength of these verb biases determines the likelihood that structural alternatives will be selected when the verb is used. Therefore, verbs without a significant preference for one sentence structure will support the selection of both alternatives to a similar degree, which leads to syntactic flexibility in the grammatical encoding system.

In Experiment 1, the frequency of the first object noun (N1) was manipulated (high vs. low frequency) in addition to syntactic flexibility. The second object noun (N2) was the same in each sentence frame across conditions and had low frequency (see 2a and 2b).

(2a) *De ober serveert/schotelt de monarch het feestmaal voor* (low frequency N1).
       “The waiter serves/dishes out the monarch the banquet.”
(2b) *De ober serveert/schotelt de koning het feestmaal voor* (high frequency N1).
       “The waiter serves/dishes out the king the banquet.”

In Experiment 2, we manipulated the frequency of the second object noun (N2; high vs. low frequency). The first object noun always had high frequency (see 3a and 3b).

(3a) *De ober serveert/schotelt de klant het feestmaal voor* (low frequency N2).
       “The waiter serves/dishes out the customer the banquet.”
(3b) *De ober serveert/schotelt de klant de maaltijd voor* (high frequency N2).
       “The waiter serves/dishes out the customer the meal.”

By examining how both frequency manipulations affected speech onsets, we can make inferences about the lexical planning scope for sentences with a high and low degree of syntactic flexibility. There are a number of different possibilities. First, speakers may engage in strictly incremental planning and prepare only the verb when they initiate their utterance (Recall that the subject noun phrase was provided at the beginning of the trial, and the verb was the first word the speakers had to produce). If this is true, there should be no effect of noun frequency at all. If speakers consistently encode their utterance up to and including the first noun before speech onset, there should be a main effect of N1 frequency, with faster onsets for utterances starting with high frequency than lower frequency nouns. If their lexical planning scope reaches, by default, up to the second noun, we expect to find a main effect of N2 frequency. More interestingly, if planning scope changes with the syntactic flexibility of the verb, there should be an interaction between noun frequency and syntactic flexibility (i.e., weak vs. strong verb bias). In line with findings of Myachykov et al. (2013) we hypothesized that syntactic flexibility would expand planning scope and that noun frequency would only have an effect on speech onsets in sentences with verbs that have no significant bias toward one syntactic frame (i.e., under high syntactic flexibility).

In addition to linguistic factors, we also examined the effect of variations in the speakers’ speed of initiating utterances on their planning scope. As noted, in studies by Wagner et al. (2010) and Wheeldon et al. (2013) speakers with long utterance onset latencies were found to have a broader planning scope than speakers with shorter latencies. We examined whether this would also be the case in the current study by computing the participants’ average speech onset latency on filler trials and adding average speech rate as a factor to a model predicting sentence onsets. By using production speed as a continuous predictor, we avoided the use of a median split procedure and obtained a more fine-grained measure of production speed.

As noted, we used the RSVP paradigm to elicit utterances with fixed wording (verbs and nouns) and structure. In this paradigm, participants are presented with a sentence in a word-by-word fashion at a high speed (100 ms per word). Subsequently they perform a short distractor task, and then see a sentence preamble (in our case the subject noun phrase), which they have to complete to form the presented sentence. It is assumed that the fast presentation of the sentence and the intervening distractor task lead to the formation of a conceptual representation of the sentence, the wording of which has to be reconstructed during later recall (Potter and Lombardi, 1990). Thus, as in everyday speech production, a conceptual message needs to be translated into a sequence of words (e.g., Bock and Levelt, 1994).

Potter and Lombardi (1990) tested their paradigm by presenting five lure words prior to the recall phase (i.e., the sentence preamble). On half of the trials, one of the lure words was conceptually related to one of the words in the to-be-recalled target sentence. After the word list, one probe word (never the conceptually related lure) was presented and participants had to judge whether this word had been part of the previously presented word list. Potter and Lombardi (1990) found that during recall, participants tended to exchange target words (verbs and nouns) for lures, but only when they were in line with the conceptual message conveyed by the target sentence. Verb exchanges even occurred when the categorization frames of the intruding verb were not compatible with the surface structure of the presented target sentence. Participants restored the grammaticality of the sentences by using a frame congruent with the selected verb (Lombardi and Potter, 1992). These results suggest that the RSVP paradigm indeed taps sentence reconstruction process rather than retrieval of an episodic memory representation of the (linearly ordered) surface structure.

The RSVP paradigm has later been used in several sentence production studies examining constraints on structural priming (e.g., Chang et al., 2003; Griffin and Weinstein-Tull, 2003; Konopka and Bock, 2009; Tooley and Bock, 2014). Here RSVP prime trials preceded structurally matching or mismatching target trials. Priming was measured as the extent to which speakers re-used the prime trial structure on target trials. Critically, the paradigm produced priming effects that were comparable in magnitude to priming effect in picture description tasks (Chang et al., 2003). This finding further supports the view that the RSVP task taps structural mechanisms of sentence production.

However, there are also obvious differences between sentence recall via the RSVP paradigm and everyday sentence production. Most importantly, the participants do not generate the message based on the thoughts they wish to express, but instead read a sentence and store its content in working memory. Based on this memory representation, the sentence has to be regenerated. The role of verbal working memory in the RSVP task manifests itself in the finding that sentence recall is often (near) verbatim. Potter and Lombardi (1998) explain the verbatim recall with the fact that speakers are likely to re-use the recently activated lexical entries, but stress that these entries are unordered in memory. Hence, during reconstruction in the recall phase, regular sentence production mechanisms are used to linearize lexical items retrieved from memory. Studies of the relationship between verbal working memory and language production are consistent with this view (Bock, 1996; Acheson and MacDonald, 2009a,b; Slevc, 2011).

In sum, RSVP offers a way of studying the generation of sentences that are otherwise not easy to elicit. Although the paradigm has its shortcomings, previous studies have shown that it can be used to tap certain aspects of normal sentence production. For the present purposes it is most important that the way retrieved lexical items are combined into sentences must be based on the participants’ general lexico-syntactic knowledge and their prior linguistic experience. We are interested in the processes involved in the retrieval of the verb and its arguments. Retrieval of a weak bias verb should result in the automatic activation of two equipotent syntactic frames (high syntactic flexibility), whereas retrieving a strong bias verb should lead to the activation of one dominant syntactic frame (low syntactic flexibility). By varying the frequency of the post-verbal nouns along with the syntactic flexibility of the verb and examining the consequences for the verb onset times, we investigate how syntactic flexibility influences the activation of upcoming lexical material prior to speech onset.

## EXPERIMENT 1

We investigated the effects of verb bias and noun frequency on speakers’ planning scope when producing dative verb phrases during sentence recall. In addition, we examined the role of participants’ response speed in explaining individual differences in advance planning. We used an RSVP paradigm to elicit sentences with fixed wording and structure: participants constructed dative sentences from a preamble (the first noun phrase, e.g., *The jeweler*) after a rapid word-by-word presentation of the entire sentence (*The/jeweler/sells/the/necklace/to/the/grandmother*). Without repeating the preamble, participants started their utterance by producing the verb, which could have a strong or a weak bias toward one dative alternative.

In addition to varying verb bias, we also manipulated the frequency of the first noun following the verb (i.e., the direct object in PO datives, and the indirect object in DO datives) The second noun was the same for each sentence frame across conditions and had low frequency. Consequently, noun frequency differences were always congruent with the verb’s preference and the presented sentence structure. Here we focus only on facilitatory or interfering effects of noun frequency on sentence production. In line with earlier findings, we expect an extension of planning scope, indexed by an effect of N1 frequency, only for sentences with weak bias verbs. In other words, we expect an interaction between verb bias and noun frequency difference on the RTs.

### METHOD

#### Participants

Thirty-six adult native speakers (ages 18–30 years) of Dutch gave informed consent and participated in the experiment for payment. All participants had normal or corrected-to-normal vision. Consent for conducting the study had been obtained from the Ethics Board of the Social Sciences Faculty of the Radboud University Nijmegen.

#### Materials

Dative verbs with weak and strong biases toward the prepositional dative and DO dative structures were selected based on a corpus analysis of the Dutch dative alternation (Colleman, 2009). In this corpus analysis, collostructional strength was identified for 252 alternating dative verbs. Collostructional strength is the degree of association between one lexical item (in this case a verb) and two or more functionally similar abstract constructions. The degree of association is based on the frequencies of the verb occurring in each of these constructions and on the overall frequencies of the construction in the corpus and is computed using the Fisher exact test. An index of distinctive collostructional strength was calculated as -log(Fisher exact, 10). The higher the index, the stronger the preference is of the verb for the construction. For example, *give* has a strong preference for the DO structure and *show* has a weak preference, with collostructional strengths of 5.56 and 0.27, respectively. Although *give* is an example of a high frequency verb, degree of strength is not correlated with lexical frequency. The corpus study revealed a wide range of collostructional strength for verbs preferring DO and PO constructions, with PO dative preferring verbs displaying a wider range (0.17–69.07) than DO dative preferring verbs (0.16–40.8).

A total of 28 verb pairs with low (*M* = 0.57, SD = 0.28) and high (*M* = 13.35, SD = 10.90) collostructional strength were selected from Colleman (2009), resulting in a weak bias and a strong bias set (e.g., *serveren* “serve” and *voorschotelen* “dish out”). For each verb pair, one sentence was constructed which could accept both verbs (e.g., *De ober serveert de klant de maaltijd* “The waiter serves the customer the meal” vs. *De ober schotelt de klant de maaltijd voor* “The waiter dishes out the customer the meal”) in the sentence frame (DO or PO) of the verb’s preference. Sentences consisted of one main clause and were written in the present tense. Verb bias conditions and dative structures were matched for verb log lemma frequency, syllable count and separability (CELEX Lexical Database, Baayen et al., 1995). Separability refers to the possibility of separating the verb core and its particle, as in for example *terugbetalen* “pay back.” Dutch has many separable verb and two placement options for the particle in PO datives: before or after the canonical position of the indirect object. In our experimental stimuli the particle always preceded the indirect object, e.g., het kind betaalt het geld terug aan de moeder “the child pays the money back to the mother.” Verb lemma frequency was uncorrelated to verb collostructional strength (*r* = 0.18). **Table 1** shows the descriptive statistics for the verbs organized by structural preference.

**Table 1:** Descriptive statistics for DO and PO dative preferring verbs used in Experiment 1.

		Collostructional strength	Log lemma frequency	Log N1 frequency	Log N2 frequency
**Verb preference**				
DO dative	Average	6.83	1.38	1.44	0.69
	SD	10.32	0.73	0.69	0.21
	Range	0.16–41	0–3.11	0.30–2.66	0.30–1.00
PO dative	Average	7.09	1.08	1.30	0.56
	SD	9.71	0.69	0.60	0.20
	Range	0.17–33	0.0–2.64	0.30–2.44	0.30–0.90

*Collostructional strength represents the bias for a DO structure for DO preferring verbs and the bias for a PO structure for PO preferring verbs. Log lemma frequency refers to the logarithmic frequency of the verb lemma. Log N1 and Log N2 frequency refer to the logarithmic frequency of the first and second post-verbal noun in the target sentence for each verb type, respectively.*

In half of the items the first noun had high frequency (*M* = 1.94, SD = 0.34) and in the other half of the items it was low in frequency (*M* = 0.81, SD = 0.29). Nouns were matched on other characteristics affecting lexical accessibility: number (plural vs. singular), length, number of syllables, and animacy. The experiment used a 2 (Verb bias) × 2 (N1 frequency) within-participant and within-item factorial design. Four lists of stimuli were created to counterbalance verb bias and N1 frequency, so that each item appeared in a different condition in each list (see Appendix A for a complete list of the stimuli used in the experiment). Within lists, there were seven items in each of the four conditions. In addition, there were 10 practice items and 84 filler items used to separate target items.

#### Norming

We carried out a norming study to evaluate whether target frames carrying different noun combinations and verbs were equally plausible. 40 participants who did not participate in the main experiments were asked to rate the plausibility of the sentences on a scale from 1 (implausible) to 7 (very plausible). Items were randomly assigned to one of four item lists, such that each sentence frame appeared in all four conditions across lists and each verb appeared exactly once per list. **Table 2** shows a summary of the norms for the selected sentence frames per condition.

**Table 2:** Mean plausibility ratings and standard deviations (in parentheses) for strong and weak bias verbs by sentence structure and N1 frequency (High N1 vs. Low N1).

	DO dative		PO dative	
Verb bias	High N1	Low N1	High N1	Low N1
Strong bias	5.97 (1.48)	5.94 (1.45)	5.61 (1.61)	5.25 (1.97)
Weak bias	5.82 (1.48)	6.05 (1.19)	5.52 (1.65)	5.30 (1.83)

Since verbs always showed a bias for the structure they occurred in and noun frequency differences were congruent too, we expected high plausibility ratings across all conditions and sentence types. As expected, ratings did not differ across verb bias *t*(1,27) = 0.39 or noun frequency conditions [*t*(1,27) = 0.57]. More importantly, there was no interaction between verb bias and N1 frequency [*F*(1,26) = 1.06]. DO datives were rated as more plausible than PO datives [*F*(1,26) = 4.57, *p* < 0.05]. This difference might be due to the fact that DO datives occur more often in Dutch than PO datives (e.g., Colleman, 2009).

#### Procedure

Participants were randomly assigned to one of the four item lists. Instructions for the experiment appeared on the screen and participants received 10 practice sentences before the experiment started. Sentences were presented in RSVP. The sequence of events for each trial is illustrated in **Table 3** (adapted from Konopka and Bock, 2009). The experiment was programmed using Presentation software (Version 16.3, ).

**Table 3:** Sequence of events per trial.

Duration	Event (example)
200 ms	+
100 ms	De
100 ms	ober
100 ms	serveert
100 ms	de
100 ms	maaltijd
100 ms	aan
100 ms	de
100 ms	klant.
100 ms	##########
533 ms	4 5 2 9 1
100 ms	[screen blanked]
500 ms	twee
10 ms	[screen blanked]
5000 ms (max)	Nee Ja
500 ms	or
Utterance onset	De ober … .

After presentation of a 200 ms fixation cross, participants read a sentence, which was presented one word at a time. Word presentation time was 100 ms, similar to earlier English RSVP studies. Although average word length in Dutch is higher than in English (Hagoort and Brown, 2000), a pilot study revealed that participants could process the words at the presentation time of 100 ms. Participants were instructed to read the sentence silently and to remember the content (i.e., the message) as they would have to reproduce it later.

They then performed a distractor task, in which they first saw a display of five digits and then had to judge whether a digit (written out in letters: e.g., *twee* “two”) had been part of this array of five digits. They responded by pressing the left (*yes*) or right (*no*) mouse button and had a maximum of 5 s to do so. They were given immediate feedback in the form of a happy face for a correct answer and a sad face for an incorrect answer. On 50% of the trials the correct answer to the distractor task was “*yes*” and on the remaining half the answer was “*no*.” On critical trials the correct answer to the distractor task was always “*yes*,” while on filler trials this could vary.

After the distractor task, participants were prompted to reproduce the sentence they had read at the beginning of the trial. The subject noun phrase of a sentence appeared on the screen (e.g., *The waiter*) and participants were asked to complete the sentence with the verb, direct object, and indirect object (i.e., without reading the subject noun phrase of the sentence out loud). They were instructed to do so as quickly as they could without making any mistakes or producing disfluencies. They then pressed a button to proceed to the next trial. Responses were recorded and the speech output was transcribed by the experimenter oﬄine. Later, speech onsets were measured manually in Praat (Boersma and Weenink, 2013).

Participants were debriefed at the end of the experimental session about the goal of the experiment. They were also asked to describe which strategy they used to remember the sentences and to what extent they remembered the exact form of the sentences. In line with findings from sentence recall studies (e.g., Bock and Brewer, 1974; Potter and Lombardi, 1990, 1998), participants reported that they often tried to reconstruct the surface structures from memory of sentence meanings.

#### Scoring and analysis

Utterances were scored as having either DO dative or PO dative syntax (e.g., *proposes the professor the plan*; *proposes the plan to the professor*). Utterances with intransitive syntax or other constructions were excluded, as were sentences with repeated sentential subjects (e.g., *The student proposed...)*, omitted direct or indirect objects (e.g., *pays back the mother*), verb substitutions (e.g., *asked* instead of *proposed*), or noun substitutions (e.g., *manuscript* instead of *plan*). Additionally, utterances following a wrong answer to the distractor task were also excluded. This was done to control for possible effects of response feedback in the distractor task on subsequent response latencies.

Finally, we eliminated responses with onset latencies longer than 3000 ms or with onset latencies more than 2.5 standard deviations away from the grand mean. The final dataset consisted of 650 responses (316 PO sentences, 334 DO sentences), equivalent to nine scorable responses per participant in each verb bias condition.

All data were analyzed using R (R Development Core Team, 2013) and the R packages *lme4* (Bates et al., 2013) and *languageR* (Baayen, 2008b). Analyses on error rates were carried out with mixed logit models (coefficients are given in log-odds). Onset latencies (RTs) were analyzed with linear mixed effects models (coefficients are given in milliseconds). Model factors included Verb bias, N1 frequency, and Sentence structure as categorical factors, after they were centered. In all analyses, we used a backward elimination procedure, starting from an initial model containing all experimental factors and their interactions and random by-subject and by-item intercepts (Baayen, 2008a; Baayen et al., 2008; Jaeger, 2008). Non-significant effects were removed, starting from the highest-order interactions going back to a basic additive model with only main effects. For the remaining fixed effects structure, random slopes were included where mentioned; they were added only if they improved model fit as indicated by likelihood ratio tests (models with maximal random structures showed similar results and are therefore not listed; cf. Barr et al., 2013). Since MCMC sampling is not implemented in *lme4* for linear mixed effects models with random effects, *p* values reported in the results were computed based on the *t*-distribution using the Satterthwaite approximation in the *lmerTest* package (Baayen et al., 2008; Kuznetsova et al., 2013).

### RESULTS

We report results of three sets of analyses. First we examined error rates across Sentence structures, N1 frequency and Verb bias conditions in the full dataset (*n* = 1008). Responses were coded as errors when they contained word substitutions or other constructions than the ditransitive, followed an incorrect answer to the distractor task, and when onset latencies exceeded the outlier threshold. In a second analysis, we tested whether Verb bias, N1 frequency and Sentence structure predicted verb onset latency after excluding errors (*n* = 650). Finally, we examined how whether the speakers’ production speed interacted with the effects of Verb bias and/or Noun frequency.

#### Error rates

**FIGURE 1:**
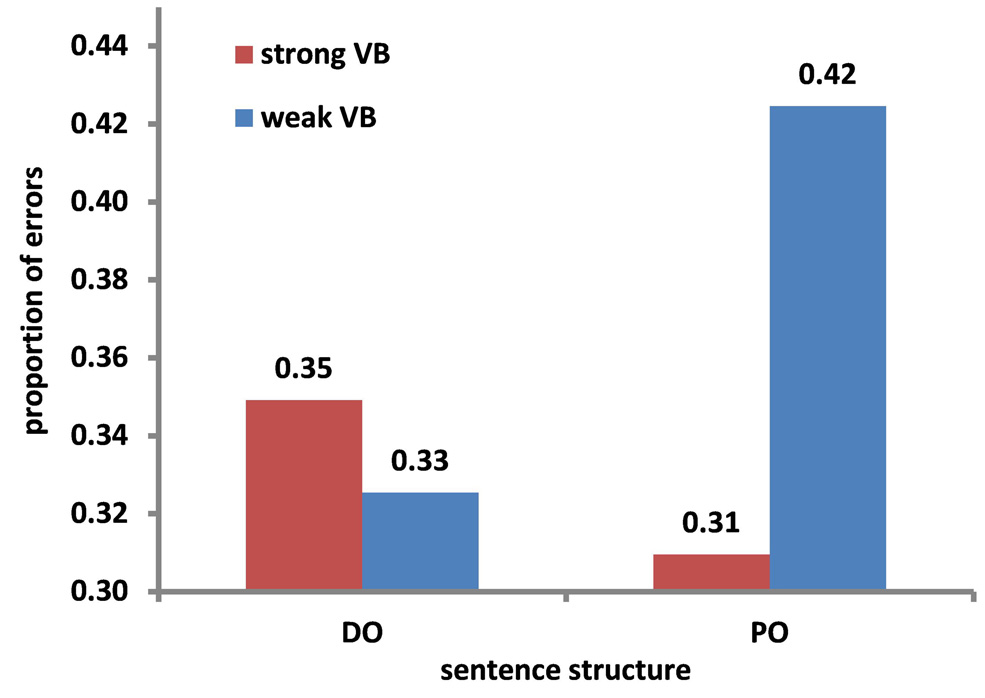
**Proportion of errors as a function of verb bias (strong VB vs. weak VB) and sentence structure (DO vs. PO)**.

Speakers’ accuracy in reproducing target sentences with the correct wording and structure was predicted by N1 frequency: speakers were more likely to correctly reproduce a sentence when the first noun had high frequency. Furthermore, there was an interaction between Verb bias and Sentence structure (respectively β = -0.36, SE = 0.14, *z* = -2.51 and β = -0.70, SE = 0.29, *z* = -2.44). **Figure 1** depicts the interaction: whereas error rates were higher for PO structures with weak bias verbs than strong bias verbs (β = -0.57, SE = 0.20, *z* = -2.91), there was no effect of verb bias in DO structures (*z* = 0.59).

#### Verb onsets

Unlike error rates, verb onsets did not show an effect of N1 frequency (*t* = -0.71). Verb bias and Sentence structure did not produce any main effects or interactions either (*t*s < -0.63).

#### Fast and slow speakers

To examine how differences in speech onset latencies were related to the performance on the RSVP task, we added average production speed to a model predicting verb onsets from Sentence structure, N1 frequency and Verb bias. To this end, we measured onset latencies on the filler trials as a neutral index of production speed. For the scoring of the filler trials we used the same criteria as for target trials: responses were excluded that (a) did not have the correct wording or structure, (b) followed a wrong answer to the distractor task, or (c) had onset latencies longer than 3000 ms and onsets more than 2.5 standard deviations away from the grand mean. Based on the remaining trials (81% of all trials, equivalent to 61 trials per participant on average) we computed the average production speed per participant. Adding this continuous factor to the full model predicting verb onsets yielded a main effect of Production speed (β = 953.49, SE = 132.65, *t* = 7.19, in a model with by-item random slopes for Production speed). There were no interactions between Production speed and the experimentally manipulated factors.

### DISCUSSION

The results of Experiment 1 only show suggestive evidence of extended planning as a result of increased syntactic flexibility. Results of the analysis on error rates suggest that planning was harder for sentences featuring weak bias verbs than for those with strong bias verbs, but only in PO datives.

One reason why syntactic planning of PO and DO datives may differ is that producing a PO structure involves more word ordering choices. First of all, speakers may choose to leave out the indirect object—which is the reason some theories regard PO structures as transitive with an optional adjunct (Dowty, 2003; Brown et al., 2012)—leading to the production of an ordinary transitive with only one obligatory argument (e.g., *submits the plan* instead of *submits the plan to the professor*). Secondly, placement of the verb particle for separable verbs [e.g., *terugbetalen* (pay back)] is also flexible for PO datives. Unlike English, Dutch has two options for placing the verb particle in a PO dative: (a) before the indirect object and (b) after the indirect object. These additional word ordering options add yet another degree of flexibility to the production of PO structure. In addition, the PO dative is less common than the DO dative (31 vs. 69% in Colleman, 2009). Altogether, the increased difficulty of structural processing for PO datives may have led speakers to build PO structures more incrementally than DO structures (Konopka, 2012). Consequently, any effect of syntactic flexibility in PO datives may not be visible at sentence onset, but only in errors. Conversely, DO datives are the easier structures and therefore speakers are not bound to a strictly incremental production strategy. Here, syntactic flexibility can have an effect on sentences initial processing load. Indeed, DO sentences featuring a strong bias verb (*M* = 751 ms, SD = 249 ms) had numerically shorter onset latencies than DO sentences with a weak bias verb (*M* = 777 ms, SD = 266 ms), but this difference was not reliable (*t* = 0.82).

The second experimental manipulation, N1 frequency, only showed an effect on error rates: Sentences with a high frequency N1 were better remembered and therefore were produced with fewer errors than sentences with a low frequency N1. The absence of N1 frequency effects on onset latencies may indicate that the first noun was not planned at verb onset. However, it could also indicate that planning scope incorporates not only the first noun, but a wider range of words. Consequently, sentence initiation times may depend on the time needed to prepare both N1 and N2, especially in syntactically flexible sentences where planning scope is hypothesized to be broader than in inflexible sentences. Since the frequency of N2 was always low in Experiment 1 so that N2 required longer preparation time than N1 (e.g., Miozzo and Caramazza, 2003), any facilitating effect of high frequency N1 might have been concealed in sentences featuring weak-bias verbs.

This hypothesis can be put to test by evaluating the effect of N2 frequency (as a between-item factor) on onset latencies for syntactically flexible sentences. If the lexical item with the longest preparation time determines the sentence onset latency in syntactically flexible sentences, then the frequency of N2 should be able to predict verb onsets in weak-verb bias sentences. Although the frequency of the two nouns in an item was carefully controlled, there were differences in N2 frequency *between* items (ranging from 0.30 to 1). Adding N2 frequency as a continuous factor to a linear mixed effects model predicting verb onsets, yielded a significant interaction between Sentence structure, Verb bias, and N2 frequency (β = -243.61, SE = 87.41, *t*= -2.79). Examining this interaction more closely revealed that the interaction between Verb bias and N2 frequency was especially apparent in PO datives (β = 437.20, SE = 126.26, *t*= 3.46) and not in DO datives (*t*= -0.83). When producing PO datives, speakers were faster to initiate sentences featuring weak bias verbs as N2 frequency increased (β = -323.16, SE = 150.06, *t*= 3.46). In contrast, N2 frequency did not produce an effect in sentences with strong bias verbs (*t*= 0.89). Taken together, these results provide an explanation for the absence of an interaction between Verb bias and N1 frequency in Experiment 1: any effect of N1 frequency on onset latencies was disguised by the parallel retrieval of the lower frequency N2.

To obtain further experimental support for the possibility that planning scope in syntactically flexible sentences includes N2, we carried out a second experiment in which we manipulated the frequency of the second noun (the direct object in DO structures and the indirect object in PO structures), while keeping N1 frequency constant, i.e., all N1s had high frequency.

## EXPERIMENT 2

The goal of Experiment 2 was to test whether syntactic flexibility could lead to an increased grammatical planning scope by manipulating the frequency of N2. We hypothesize that syntactic flexibility expands planning scope up to and including N2 during the recall of dative verb phrases. We thus expect to see an influence of N2 frequency on onset latencies only for syntactically flexible sentences (i.e., the weak verb bias condition). In other words, there should be an interaction between Verb bias condition and N2 frequency.

### METHOD

#### Participants

A different group of 36 adult native speakers (ages 18–30 years) of Dutch gave informed consent and participated in the experiment for payment. All participants had normal or corrected-to-normal vision.

#### Materials

Verb pairs were mostly similar to those used in Experiment 1. Four new verb pairs were added. **Table 4** shows the descriptive statistics for the set of 32 verb pairs.

**Table 4:** Descriptive statistics for DO dative and PO preferring verbs used in Experiment 2.

		Collostructional strength	Log lemma frequency	Log N1 frequency	Log N2 frequency
**Verb preference**					
DO dative	Average	6.30	1.30	1.69	1.05
	SD	9.80	0.74	0.47	0.70
	Range	0.16–41	0–3.11	0.78–2.66	0–2.40
PO dative	Average	7.39	1.07	1.60	0.93
	SD	10.22	0.68	0.47	0.67
	Range	0.17–41	0–2.64	0.78–2.44	0–2.37

*Collostructional strength represents the bias for a DO structure for DO preferring verbs and the bias for a PO structure for PO preferring verbs. Log lemma frequency refers to the logarithmic frequency of the verb lemma. Log N1 and Log N2 frequency refer to the logarithmic frequency of the first and second post-verbal noun in the target sentence for each verb type, respectively.*

In half of the items the second noun had low frequency (*M* = 0.42, SD = 0.28) and in the other half of the items the frequency of the second noun matched the first noun’s frequency (i.e., high frequency: *M* = 1.57, SD = 0.44). As in Experiment 1, nouns were matched on other characteristics affecting lexical accessibility. By varying the frequency of the second noun, the direction of frequency differences was always congruent with verb bias. Hence, in sentences with PO biasing verbs, the direct object was more frequent than, or equally frequent as, the indirect object. In sentences with DO biasing verbs, the indirect object was more frequent than, or equally frequent as, the direct object (see example 3a and 3b in the Introduction).

The experiment used a 2 (Verb bias) × 2 (N2 frequency) within-participant and within-item factorial design. Four lists of stimuli were created to counterbalance verb bias and noun-frequency conditions, so each item appeared in a different condition in each list (see Appendix B for a complete list of the stimuli used in the experiment). Within lists, there were eight different items in each of the four conditions. 94 fillers were included to separate target items, with 10 used at the beginning of the experiment as practice items.

#### Norming

A new norming study with 60 participants confirmed that target frames carrying different noun combinations and verbs were equally plausible. **Table 5** shows a summary of the norms for the selected sentence frames per condition.

**Table 5:** Mean plausibility ratings and standard deviations (in parentheses) for strong and weak bias verbs by sentence structure and N2 frequency (High N2 vs. Low N2).

	DO dative		PO dative	
Verb bias	High N2	Low N2	High N2	Low N2
Strong bias	5.42 (1.80)	5.85 (1.53)	5.55 (1.71)	5.34 (1.83)
Weak bias	5.27 (1.88)	5.34 (1.73)	5.18 (1.84)	5.34 (1.73)

Ratings did not differ across Noun frequency conditions and Sentence structures (*ts*< 1.31). Importantly, there was no interaction between Verb bias and N2 frequency [*F*(1,30) = 0]. However, weak verb bias sentences were rated to be slightly less plausible than sentences with strong bias verbs, *t*(1,31) = 1.82, *p* = 0.08.

#### Procedure

The same procedure was used as in Experiment 1.

#### Scoring and analysis

Scoring was the same as in Experiment 1. Again, analyses were carried out using mixed logit models and linear mixed effects models. Fixed factors included Verb bias, N2 frequency, and Sentence structure. Additional analyses were carried out including the predictor Plausibility (according to the item norming) to assess the effects of the experimentally manipulated factors above and beyond the effect of the plausibility ratings. These analyses confirmed the pattern of results obtained from analyses using only experimentally manipulated factors and are therefore not reported.

### RESULTS

#### Error rates

Speakers made more errors in reproducing sentences with weak bias verbs (*M* = 0.36, SD = 0.48) than strong bias verbs (*M* = 0.31, SD = 0.46), but the difference was only marginally significant (β = -0.25, SE = 0.13, *z* = -1.90, *p* = 0.06). Error rates did not differ across Noun frequency conditions or Sentence structures (*z*s < 1.17).

#### Verb onsets

Onset analyses were carried out on trials where speakers produced sentences with the intended structure (*n* = 754), making use of linear mixed effects models. Verb bias, N2 frequency and Sentence structure were fixed predictors in these models.

**FIGURE 2:**
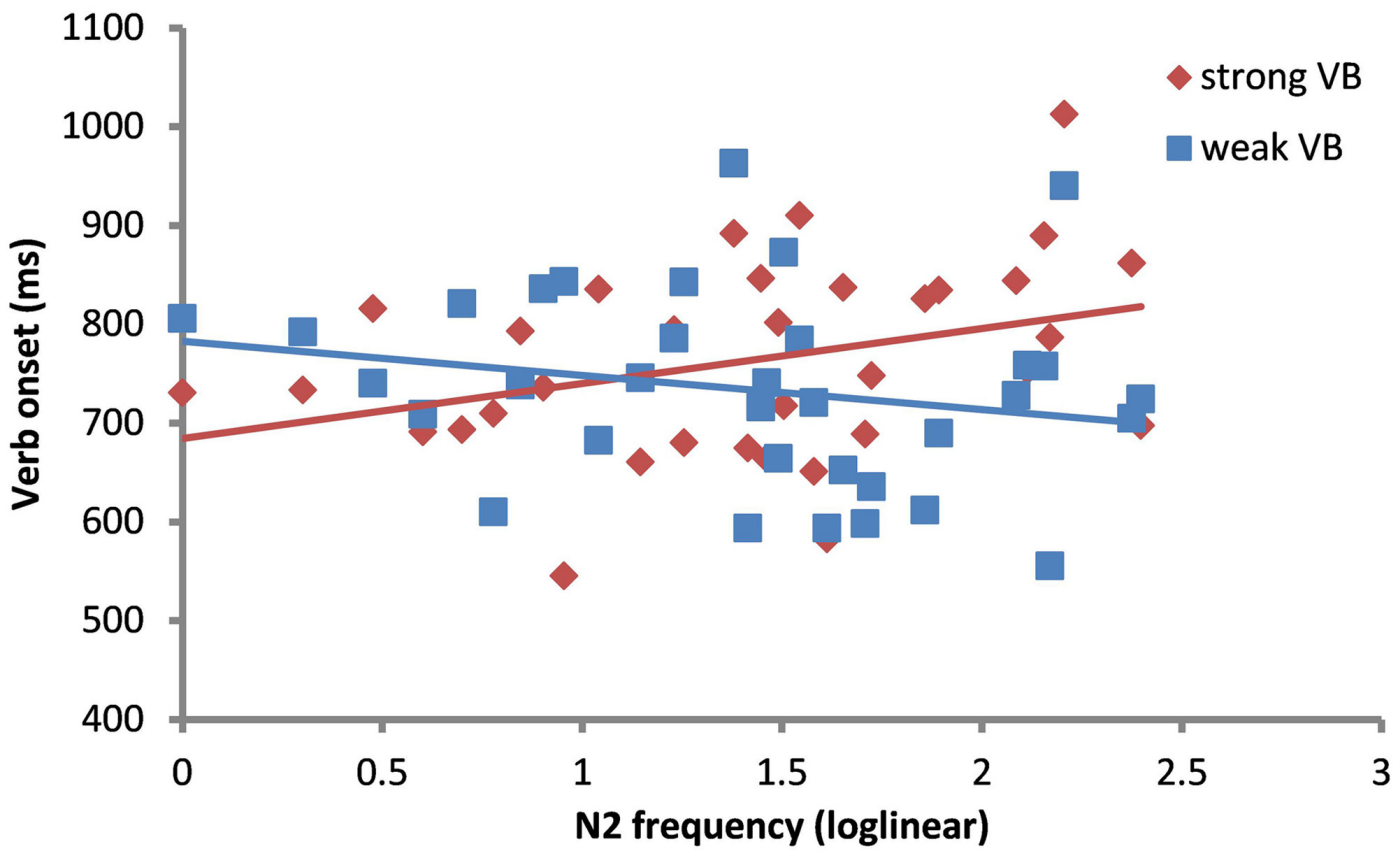
**Scatterplot showing joint effects of N2 frequency and Verb bias on Verb onset latencies, collapsing across sentence type**.

In line with our predictions, we found a significant interaction between Verb bias and N2 frequency (β = -55.43, SE = 27.99, *t* = -1.98, *p*< 0.05). Speakers were faster to produce weak-verb bias sentences with a high frequency N2 than with a low frequency N2. In sentences with strong bias verbs, onsets were not predicted by N2 frequency (*t* = -0.80). To examine this effect more closely, we ran a second model with N2 frequency as a continuous predictor. **Table 6** summarizes the fixed effects of the best model fit. In this model, Verb bias showed a (now stronger) interaction with N2 frequency (*p*< 0.01). **Figure 2** shows the interaction between Verb bias and N2 frequency.

**Table 6:** Summary of fixed effects in the linear mixed effects model predicting verb onset latencies in Experiment 2.

Predictor	Coefficient	SE	*t*	*Pr*(>|*t*|)
(Intercept)	765.38	24.47	31.27	<2e-16
Structure	-29.45	19.03	-1.55	0.13
Verb bias	15.46	15.93	0.97	0.34
N2 frequency (continuous)	- 6.01	12.88	-0.47	0.64
Verb bias by N2 frequency	70.72	23.82	2.97	<0.01

*N = 754, log-likelihood = -5044. By-subject random slopes are included for the interaction between Verb bias and N2 frequency.*

Speakers were faster to initiate weak-verb bias sentences featuring nouns with a high frequency N2 than a low frequency N2 (β = -39.63, SE = 18.04, *t* = -2.20). In contrast, N2 frequency did not produce an effect in sentences with strong bias verbs (*t* = 1.53).

#### Fast and slow speakers

**FIGURE 3:**
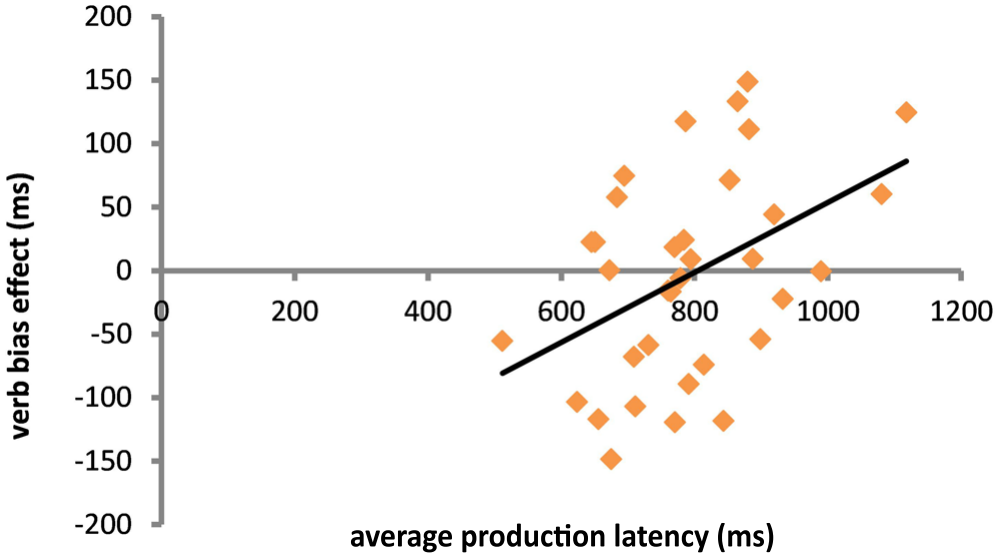
**Scatterplot of the relationship between production latency and the magnitude of the verb bias effect (weak bias RT – strong bias RT).** For this plot, outliers (*n* = 2) based on extreme values of Cook’s distance were excluded.

The fact that by-subjects random slopes for the interaction between Verb bias and N2 frequency improved model fit in a model predicting verb onsets (see above) already suggests that there was substantial subject-level variability in the strength of this interaction. One possible source for these individual differences is production speed (Wagner et al., 2010). Therefore, we measured average production latencies on the filler trials per participant as an index of production speed. After excluding incorrect responses and outliers as on the target trials (see above), we added this factor to a model predicting verb onsets from Verb bias, N2 frequency (continuous), and Sentence structure. The final model included a significant two-way interaction between Verb bias and Production speed (β = -0.29, SE = 0.11, *t* = -2.61). **Figure 3** shows the interaction between Verb bias and Production speed.

A longer average speech onset latency on the filler items was associated with a larger verb bias effect on experimental items, such that sentences with weak bias verbs were produced more slowly than sentences with strong bias verbs. High production speed on filler trials was associated with an effect of verb bias in the opposite direction; weak verb bias sentences were initiated slightly faster than strong verb bias sentences.

There was a trend toward a three-way interaction between Verb bias, N2 frequency and Production speed (β = 0.27, SE = 0.16, *t* = 1.65, *p* = 0.10); in the weak bias condition, slower production speed led to a larger delay in producing utterances with low frequency N2 compared to a high frequency N2. In utterances with strong bias verbs there were no differences in onset times for low and high frequency N2s, nor was there a relationship with production speed.

### DISCUSSION

The results of Experiment 2 provide evidence for extensive planning at the lexical level in sentences with syntactic flexibility. The significant interaction between N2 frequency and Verb bias suggests that primarily in sentences with weak bias verbs, lexical planning scope included N2. This effect was especially apparent in slower speakers who showed delayed onsets for weak-verb bias sentences with low frequency N2s relative to weak-verb bias sentences with high frequency N2s.

## GENERAL DISCUSSION

The aim of this study was to examine the influence of the availability of multiple syntactic frames (i.e., syntactic flexibility), and hence multiple placement options of post-verbal material, on the lexical planning scope during recall of dative verb phrases. Syntactic flexibility was varied by using verbs with different degrees of bias toward each dative alternative. In addition, lexical accessibility was varied by using post-verbal nouns with low and high frequency. Assessing the effect of increased lexical accessibility on speech onsets allowed us to make inferences about the scope of lexical planning during sentence recall. In Experiment 1, the frequency of the first post-verbal noun was varied (i.e., the direct object in PO and the indirect object in DO datives). Results only provided suggestive evidence that planning scope was wider for weak than strong bias verbs.

In Experiment 2, we manipulated the frequency of the second post-verbal noun (i.e., the indirect object in PO and the direct object in DO datives). Results provided evidence for lexical planning up to and including the second post-verbal noun in syntactically flexible sentences. A significant interaction was found between verb bias and N2 frequency; N2 frequency only mattered during the recall of weak bias sentences. Weak bias sentences with a high frequency N2 were initiated faster than sentences with a low frequency N2. There was substantial inter-subject variability and the interaction between verb bias and N2 frequency was primarily driven by the speakers with longer average speech onset latencies on filler trials—the slow speakers. Previous research has shown that slow speakers (i.e., speakers who initiate their utterances with relatively long latencies) engage in more extensive advance planning than fast speakers (Wagner et al., 2010; Wheeldon et al., 2013). The present results suggest that slow speakers may be more flexible in extending their planning scope than fast speakers. Wagner et al. (2010) found that speakers decrease their scope of planning under cognitive load induced by a secondary conceptual decision task. Hence, extensive advance planning is cognitively more demanding than piecemeal, incremental planning. Slow speakers might thus have more cognitive capacity (e.g., cognitive control), allowing them to engage in flexible advance planning than faster speakers. Further experimentation is needed to investigate this possibility.

There are two possible mechanisms through which syntactic flexibility could influence the activation of lexical material and planning scope. According to a first hypothesis, syntactic flexibility induced by weak verb bias may give rise to the activation of both post-verbal nouns in the dative verb phrase: when retrieving a weak bias verb, two dative structural alternatives featuring different object orders become activated to roughly the same degree. Consequently, both post-verbal nouns (i.e., the direct and the indirect object) may become activated, as they could both fill the position immediately after the verb. The insertion of the direct object would lead to the production of a PO dative, while the insertion of the indirect object would lead to the production of a DO dative. This hypothesis is in line with a single-stage view of grammatical encoding, suggesting that upon verb retrieval, the processor automatically tries to select the constituent immediately adjacent to the verb in the linear structure (Pickering et al., 2002; Cai et al., 2012).

On a different account, syntactic flexibility may lead to competition between abstract dative sentence frames (Ferreira, 1996; Myachykov et al., 2013; Hwang and Kaiser, 2014). During the time needed to resolve competition and select a target frame, post-verbal objects may be retrieved in parallel. In the absence of competition (i.e., in sentences with strong bias verbs), the utterance may be initiated immediately after retrieval of the verb (and possibly the first noun). This account is in line with a multiple-stage view of grammatical encoding, in which an abstract (i.e., lexically independent) hierarchical structure intermediates the mapping from functional-level input to a linear structure (e.g., Bock and Levelt, 1994).

Our data do not distinguish between these two accounts. Both predict that under syntactic flexibility N2 should be activated, be it immediately after verb retrieval as a candidate to fill the post-verb position, or during the resolution of competition between the two dative candidate frames. Importantly though, both accounts imply that upon the retrieval of a weak-bias verb, syntactic flexibility offers a speaker the choice to insert an indirect or direct object into the developing verb phrase/to construct a PO or a DO dative frame.

The process of choosing between the insertion of the direct or indirect object in the post-verbal slot may influence onset latencies for sentences with weak bias verbs in two different ways. On the one hand, frequency differences between to-be-inserted nouns may support quick settling on the “winning” syntactic alternative. On this *relative* frequency view, noun frequency differences may help the speaker in choosing a structure, by promoting the insertion of the higher frequency noun into the sentence structure first (cf. Stallings et al., 1998; Stallings and MacDonald, 2011). Consequently, if the direct object NP has higher frequency than the indirect object NP, the selection of a PO structure will be promoted, while an indirect object NP with higher frequency promotes the selection of a DO structure. Note that in both experiments, noun frequency differences were always congruent with the presented sentence structure and the preference of its verb, i.e., N1 always had higher frequency than N2. However, differences still existed in the *degree* of consistency between the presented sentence structure, its verb bias and noun frequency differences. That is, a noun ordering in which N1 has higher frequency than N2 (i.e., HF–LF ordering) is *more* consistent than an ordering with a high frequency N1 and a high frequency N2 (HF–HF, or in Experiment 1: LF–LF). Therefore, a *relative* frequency view predicts quick settling on a structure with a weak bias verb (and therefore shorter onset latencies) for (a) the high frequency N1 condition in Experiment 1 (i.e., HF–LF ordering), and (b) the low frequency N2 condition in Experiment 2 (i.e., HF–LF ordering). Although our results did not show any effect of N1 frequency, the opposite pattern was observed for N2 frequency. Noun frequency differences did not facilitate structure choice. Instead, speakers were *slower* to initiate weak-bias sentences with a HF–LF ordering than with a HF–HF ordering.

Therefore it seems that the *absolute* frequency of the nouns within speakers’ scope of planning matters for the onset latencies. On this *absolute* frequency view, structure choices are made rather independently from the lexical frequency of the nouns and effects of frequency on onset latencies only reflect the ease of retrieving the nouns that are within the scope of planning. This view thus predicts that sentences with weak bias verbs can only be initiated when the to-be-produced noun with the lowest frequency is prepared. Findings from both experiments support this view by showing that onset latencies were longer for weak-verb bias sentences with low frequency than high frequency N2s (and high frequency N1s, i.e., HF–LF ordering).

Although syntactic flexibility affected verb onset latencies, it did not affect the speakers’ production choices. In Experiment 1 and 2 together, only eight occasions of flipping (i.e., producing the alternative structure to the presented one) were found. There are two reasons why syntactic flexibility did not affect production choice. Firstly, verb biases and differences in the frequency of the first and second post-verbal noun were always congruent with the sentence structure presented to the participant. For instance, the verb *voorschotelen* “dish out” which has a strong bias toward the DO object dative, was only presented in a DO frame and with an indirect object (e.g., *king*) that was more frequent than the direct object (e.g., *banquet*). These circumstances all promoted the usage of the structure as it was presented to the participant. Only in sentences with weak-bias verbs and nouns with equal frequency (e.g., low frequency N1 with a low frequency N2 in Experiment 1 and high frequency N1 with a high frequency N2 in Experiment 2), conditions supported flipping of sentence structure–although speakers might have been primed to re-use the presented structure through the paradigm that we used. Indeed, flipping of sentence structure primarily occurred in the weak-verb bias condition (6 out of 8 occurrences) and when the frequency of N1 and N2 was (almost) equal (6 out of 8 occurrences). Secondly, as many authors have pointed out, the RSVP paradigm promotes re-usage of the same structure and lexical material (Potter and Lombardi, 1998; Chang et al., 2003; Tooley and Bock, 2014). Structural priming effects have been observed in many sentence generation studies, and these effects tend to increase in strength when prime and target share lexical content (Pickering and Branigan, 1998; Cleland and Pickering, 2003). Thus, it is not surprising to observe them in the RSVP paradigm as well.

One might see the absence of structural differences between the target sentences and the sentences participants reproduced as suggesting that the participants did not reconstruct the sentences based on the conceptual structure but instead retrieved the fully specified string of words from working memory. However, in the post-experimental debriefing, participants reported that they often forgot the precise content and wording of the sentence due to the intervening distractor task and had to reconstruct the wording from their memory of the underlying sentence meaning. The nature of their errors provides converging evidence for this observation. In both experiments, the majority of the errors (56 and 70 % of the errors in Experiment 1 and 2, respectively) were substitutions of the target verb and/or a noun with often conceptually similar words, e.g., the use of “entrepreneur” instead of “buyer.” Most importantly, it is difficult to see how the observed interaction between Verb bias and Noun frequency could arise if the linearized sequence of words were retrieved from working memory: since verbs and nouns in the target sentences were carefully matched on characteristics influencing their accessibility (e.g., frequency of the verb and non-manipulated object noun, plausibility, length, and syllable count), no systematic effects on onset latencies would be expected by a strictly episodic view. Instead, the interaction suggests that verb retrieval during recall involves the activation of associated subcategorization frames to the degree specified by the verb’s bias, which in turn influences the degree to which upcoming lexical material is (re-)activated at verb onset (cf. Melinger and Dobel, 2005).

In sum, results of Experiment 1 and 2 suggest that syntactic flexibility expands planning scope by promoting the early activation of lexical material during sentence recall. This is in line with findings from Myachykov et al. (2013), who found that speakers of a less flexible language (English) showed more strictly incremental sentence planning than speakers of a syntactically flexible language (Russian). The current study extends these findings by manipulating syntactic flexibility within one language (Dutch), using a different sentence structure (datives) and a different paradigm.

## Conflict of Interest Statement

The authors declare that the research was conducted in the absence of any commercial or financial relationships that could be construed as a potential conflict of interest.
