# Supplementary material for: Syntactic flexibility and planning scope: the effect of verb bias on advance planning during sentence recall
Source: Front Psychol. 2014 Oct 20;5:1174. doi: 10.3389/fpsyg.2014.01174 (PMC4202777; doi:10.3389/fpsyg.2014.01174)
Supplement: Supplementary file 1 [file Data_Sheet_1.DOCX]

***Supplementary material***

Syntactic flexibility and planning scope:

The effect of verb bias on grammatical advance planning

**Maartje van de Velde^1,2^*, Antje S. Meyer^1,3^**

^1^Max Planck Institute for Psycholinguistics, Nijmegen, The Netherlands

^2^Max Planck International Research School for Language Sciences, Nijmegen, The Netherlands

^3^Donders Institute for Brain, Cognition and Behavior, Radboud University, Nijmegen, The Netherlands

**Correspondence:** M. van de Velde, Max Planck Institute for Psycholinguistics, P.O. Box 310, 6500 AH Nijmegen, The Netherlands

[maartje.vandevelde@mpi.nl](mailto:agnieszka.konopka@mpi.nl)

**Appendix A**

| **verb pair (strong/weak)** | **verb pair English** | **sentence frame Dutch (*with high vs. low frequency N1*)** | **sentence frame English (*with high vs. low frequency N1*)** |
| --- | --- | --- | --- |
| **PO biased** |  |  |  |
| versturen/zenden | send | De aanklager verstuurt ***een bericht/telegram*** aan de raadsman | The client sends ***a message/telegram*** to the lawyer |
| overmaken/uitbetalen | transfer/pay out | De boer maakt ***het geld/de som*** over aan de smid | The farmer transfers ***the money/sum*** to the smith |
| schenken/cadeau doen | donate | De directeur schenkt ***het horloge/de vulpen*** aan de griffier | The director donates ***the watch/pen*** to the clerk |
| bekend maken/verklappen | reveal | De goochelaar maakt ***het geheim/de truc*** bekend aan de concurrent | The magician reveals ***the secret/trick*** to the competitor |
| slijten/verkwanselen | sell/bargain away | De juwelier slijt ***de steen/het goud*** aan de knul | The jeweler sells ***the stone/gold*** to the boy |
| uitdelen/voeren | distribute/feed | De kinderen delen ***het eten/de appels*** uit aan de geiten | The children distribute ***the food/apples*** to the goats |
| afstaan/nalaten | relinquish/bequeath | De oma staat ***de foto's/sieraden*** af aan de verwanten | Grandma relinquishes ***the photos/jewelry*** to the relatives |
| terugbetalen/noemen | pay back/mention | De ondernemer betaalt ***het bedrag/de lening*** terug aan de financier | The merchant pays back ***the amount/loan*** to the financer |
| melden/verkondigen | report/proclaim | De president meldt ***de theorie/het verzinsel*** aan de adviseur | The president reports ***the theory/fabrication*** to the advisor |
| lenen/opdragen | lend/dedicate | De schrijver leent ***het boek/debuut*** aan de fan | The author lends ***the book/debut*** to the fan |
| doorspelen/doorschuiven | pass | De spelleider speelt ***de kaarten/fiches*** door aan de amateurs | The leader passes ***the cards/chips*** to amateurs |
| voorleggen/voordragen | submit/recite | De student legt ***het verhaal/pleidooi*** voor aan de docent | The student submits ***the story/argument*** to the lecturer |
| betalen/uitbetalen | pay/pay out | De verzekeraar betaalt ***de schade/onkosten*** aan de huurder | The insurer pays ***the damages/expenses*** to the tenant |
| verlenen/bieden | give/offer | De zakenman verleent ***hulp/bijstand*** aan het oudje | The businessman gives ***help/assistance*** to the oldie |
| **DO biased** |  |  |  |
| wijzen/laten zien | point/showing | De agent wijst ***de chauffeur/bestuurder*** het sein | The officer points ***the*** ***driver/operator*** the signal |
| adviseren/aanbevelen | advise/recommend | De arts adviseert ***de patient/bejaarde*** het medicijn | The doctor advises ***the patient/senior*** the ointment |
| garanderen/bevestigen | guarantee/confirm | De bankier garandeert ***de meneer/cliente*** het rendement | The dealer guarantees ***the sir/client*** the returns |
| verbieden/weigeren | prohibit/refuse | De barman verbiedt ***het meisje/de puber*** de likeur | The bartender prohibits ***the girl/adolescent*** liquor |
| opleveren/verschaffen | provide | De loterij levert ***de leden/spelers*** tonnen op | The lottery provides ***the members/players*** thousands |
| beletten/ontzeggen | prevent/deny | De douane belet ***het gezelschap/de misdadiger*** de doorgang | Customs prevents ***the group/criminal*** the passage |
| leren/voorlezen | teach/read out | De korpschef leert ***de groep/het team*** de instructie | The police chief teaches ***the group/team*** the instruction |
| verzoeken/vragen | request/ask | De man verzoekt ***de vriend/kelner*** de gunst | The man requests ***the friend/waiter*** the favor |
| meegeven/toegooien | give along/toss | De moeder geeft ***het kind/ventje*** de peer mee | The mother gives ***the child/the little*** *boy* the pear |
| voorschotelen/serveren | dish out/ serve | De ober schotelt ***de koning/monarch*** het feestmaal voor | The waiter dishes out ***the king/monarch*** the banquet |
| bezorgen/toewerpen | deliver/throw | De postbode bezorgt ***het gezin/echtpaar*** het pakket | The postman delivers ***the family/couple*** the package |
| ontnemen/benemen | deprives/take away | De stank ontneemt ***de jongen/keizer*** de eetlust | The smell deprives ***the boy/emperor*** the appetite |
| aanraden/aanprijzen | advise/recommend | De verkoper raadt ***de jongeman/afnemer*** de stofzuiger aan | The seller advises ***the young man/customer*** the vacuum cleaner |
| geven/aanreiken | give/hand | De verzorger geeft ***de hond/aap*** het voer | The guard gives ***the dog/monkey*** the food |

**Appendix B**

| **verb pair (strong/weak)** | **verb pair English** | **sentence frame Dutch (*with high vs. low frequency N2*)** | **sentence frame English (*with high vs. low frequency N2*)** |
| --- | --- | --- | --- |
| **PO biased** |  |  |  |
| betalen/uitbetalen | pay/pay out | De bewoner betaalt de schade aan *de* ***eigenaar/verhuurder*** | The resident pays the damages to ***the owner/landlord*** |
| versturen/zenden | send | De cliënt verstuurt een bericht aan *de* ***advocaat/raadsman*** | The client sends a message to ***the lawyer/adviser*** |
| overmaken/uitbetalen | transfer/pay out | De dader maakt het bedrag over aan ***het slachtoffer/de gedupeerde*** | The offender transfers the amount to ***the victim/duped*** |
| schenken/cadeau doen | donate | De directeur schenkt het horloge aan ***de werknemer/diplomaat*** | The director donates the watch to ***the employee/diplomat*** |
| afleggen/zweren | swear | De getuige legt de eed af aan ***de jurist/rechtszaal*** | The witness swears the oath to ***the jurist/courtroom*** |
| bekend maken/verklappen | reveal | De goochelaar maakt de truc bekend aan ***de toeschouwer/toehoorder*** | The magician reveals the trick to ***the audience/hearer*** |
| terugbetalen/noemen | pay back/mention | De handelaar betaalt de kosten terug aan ***de directeur/aannemer*** | The merchant pays back the costs to ***the director/contractor*** |
| slijten/verkwanselen | sell/bargain away | De juwelier slijt de ketting aan ***de koper/diva*** | The jeweler sells the necklace to ***the buyer/diva*** |
| uitdelen/voeren | distribute/feed | De kinderen delen het brood uit aan ***de honden/zwanen*** | The children distribute the bread to ***the dogs/swans*** |
| doorspelen/doorschuiven | pass | De leider speelt de kaarten door aan ***de spelers/gokkers*** | The leader passes the cards to ***the players/gamblers*** |
| uitdelen/nalaten | distribute/bequeath     \| bequeath \|  \| \| --- \| --- \| | De opa deelt het geld uit aan ***de familie/verwanten*** | Grandpa distributes the money to ***the family/relatives*** |
| melden/verkondigen | report/proclaim | De president meldt de leugen aan ***de natie/meute*** | The president reports the lie to ***the nation/mob*** |
| lenen/opdragen | lend/dedicate | De schrijver leent het boek aan ***het meisje/de minnares*** | The author lends the book to ***the girl/mistress*** |
| voorleggen/voordragen | submit/recite | De student legt het gedicht voor aan ***de leraar/docent*** | The student submits the poem to ***the teacher/lecturer*** |
| overdragen/uitloven | transfer/offer | De winkelier draagt de prijs over aan ***het personeel/de winnaar*** | The retailer transfers the prize to ***the staff/winner*** |
| verlenen/bieden | give/offer | De zakenman verleent hulp aan ***de vriend/het oudje*** | The businessman gives assistance to ***the friend/oldie*** |
| **DO biased** |  |  |  |
| wijzen/laten zien | point/show | De agent wijst de chauffeur ***het bord/sein*** | The officer points the driver ***the sign/signal*** |
| adviseren/aanbevelen | advise/recommend | De arts adviseert de patient ***het middel/zalfje*** | The doctor advises the patient ***the remedy/ointment*** |
| verbieden/weigeren | prohibit/refuse | De barman verbiedt de leerling ***de alcohol/tequila*** | The bartender prohibits the student ***alcohol/tequila*** |
| geven/aanreiken | give/hand | De bewaker geeftt de aap ***het voer/de biet*** | The guard gives the monkey ***the food/beet*** |
| ontfutselen/aftroggelen | pilfer | De dief ontfutselt de soldaat ***het horloge/uurwerk*** | The thief pilfers the soldier ***the watch/clock*** |
| beletten/ontzeggen | prevent/deny | De douane belet de misdadiger ***de doorgang/doortocht*** | Customs prevents the criminal ***the passage/transit*** |
| garanderen/bevestigen | guarantee/confirm | De handelaar garandeert de cliënt ***het voordeel/rendement*** | The dealer guarantees the client ***the advantage/returns*** |
| leren/voorlezen | teach/read out | De lerares leert de groep het ***verhaal/relaas*** | The teacher teaches the children ***the story/tale*** |
| besparen/sparen | save | De lift bespaart de jongen ***moeite/het gedoe*** | The elevator saves the boy ***the effort/trouble*** |
| opleveren/verschaffen | provide | De loterij levert de deelnemers ***prijzen/tonnen*** op | The lottery provides the participants ***prizes/thousands*** |
| voorschotelen/serveren | dish out/ serve | De ober schotelt de klant ***de maaltijd/het feestmaal*** voor | The waiter dishes out the customer ***the meal/banquet*** |
| bezorgen/toewerpen | deliver/throw | De postbode bezorgt het gezin ***de krant/het pakket*** | The postman delivers the family ***the newspaper/package*** |
| meegeven/toegooien | give along/toss | De vader geeft het kind ***het boek/de peer*** mee | The father gives the child ***the book/pear*** |
| aanraden/aanprijzen | advise/recommend | De verkoper raadt de klanten ***het apparaat/de stofzuiger*** aan | The seller advises the customers ***the apparatus/vacuum cleaner*** |
| verzoeken/vragen | request/ask | De voorzitter verzoekt de minister om ***een verklaring/opheldering*** | The Chairman requests the Minister ***a statement/clearing*** |
| ontnemen/benemen | take away | Het hek ontneemt de jongeman ***het uitzicht/de uitkijk*** | The fence takes away the young man ***the view/lookout*** |
